# Effect of Interferon Treatment on Hearing of Patients with Chronic Hepatitis C

**DOI:** 10.4103/1319-3767.77240

**Published:** 2011

**Authors:** Abdulrahman Hagr, Dima Jamjoom, Faisal M. Sanai, Waleed Al Hamoudi, Ayman A. Abdo, Ahmed Al-Arfaj

**Affiliations:** Department of ENT, College of Medicine, King Saud University, Riyadh, Saudi Arabia; 1 Department of ENT, College of Medicine, King Saud University, Riyadh, Saudi Arabia; 2 Department of Medicine, Division of Gastroenterology & Hepatology, Riyadh Military Hospital, Riyadh, Saudi Arabia; 3 Gastroenterology Division, Department of Medicine, King Saud University, Riyadh, Saudi Arabia

**Keywords:** Hearing loss, hepatitis C, interferon, treatment

## Abstract

**Background/Aim:**

Some reports in the literature have linked interferon therapy for the treatment of hepatitis C (HCV) with hearing loss. The aim of this study has been to examine the effects of interferon therapy on hearing of patients treated for HCV.

**Patients and Methods:**

Patients were recruited according to preset inclusion criteria from two centers. All patients received standard dose pegylated interferon (PEG-IFN α-2b or α-2a) plus ribavirin (RBV). All patients had pure-tone audiometry (PTA), tympanogram and distortion-product otoacoustic emission (DPOAE) before treatment, three months after initiation of treatment, and three months after completion of treatment.

**Results:**

Twenty one patients were prospectively recruited. The mean age was 45.7 years. The male to female ratio was 1.1:1. The mean PTA was 15.9 ± 5.3 before treatment, 17.4 ± 6.1 during treatment and 16.5 ± 5.1 after treatment. The differences between pre and mid, pre and post, as well as mid and post were not significantly different (*P*>0.05) in all audiological assessments.

**Conclusions:**

Our results indicate that PEG-IFN\RBV therapy does not have any impact on the hearing thresholds of patients with HCV.

Worldwide, more than 150 million people are infected with the hepatitis C virus (HCV),[1] a number that is believed to be an underestimate of the true global prevalence of this disease.[2] Interferon-a (IFN-α) has been used to treat HCV infection since the early 1990’s, with improved outcomes in recent years following the introduction of pegylated interferons (PEG-IFN) and ribavirin (RBV) combination therapy, resulting in sustained virological response (SVR) in 60-90% of patients, depending on the viral genotype.[3] As a result, an increasing number of patients are being identified and treated for HCV using PEG-IFN \ RBV.

Interferon α is a highly pleiotropic cytokine with potent immunoregulatory, antiproliferative, differentiation-inducing, proapoptotic and antiangiogenic effects.[4] In addition, it induces proteins and enzymatic pathways that establish an antiviral state in infected and uninfected cells. IFN-α binds to its receptors at the surface of the immune cells which triggers complex and intricate effects such as class I major histocompatibility complex antigen expression, activation of effector cells as well as complex interactions with the cytokine cascade.[5] The combined therapy with PEG-IFN\RBV is known to activate the T-helper lymphocytes promoting a Th1 profile immune response as a mechanism against viruses.

Recently, sudden hearing loss has been reported in patients treated with interferon therapy.[6–15] The reported incidence of hearing loss associated with IFN-α treatment ranges from 0.1%[16] to 39.5%.[8] Interestingly, hearing loss was usually unilateral[17] which can make it insidious in onset and difficult to detect. Fortunately, most reported patients recovered after discontinuation of therapy, although some did not recover completely.[10,18] Most studies reporting this side effect were case reports or animal studies.[19] This aspect has not been studied prospectively, where systematic measurements of hearing could be undertaken to adequately address the issue. On the other hand, other studies have reported no ototoxicity at all.[20,21] Ironically, Kanemaru[22] suggested that IFN therapy may be effective and safe in the treatment of idiopathic sudden sensorineural hearing loss (ISSHL).

The current study has been performed to prospectively evaluate whether interferon therapy impacts on hearing, and if so, delineate in detail, its timing, severity, nature, and reversibility.

## PATIENTS AND METHODS

Patients with compensated chronic, treatment-naive HCV were enrolled for this study. The following inclusion criteria had to be fulfilled: age between 18 and 60 years, a positive test for anti-HCV, HCV RNA positive by PCR, and absence of contraindications for antiviral therapy. Exclusion criteria were previous history of ear disease, active auditory symptoms at the time of recruitment, decompensated cirrhosis, other causes of liver disease, autoimmune disorders and other severe comorbidities such as neoplastic, cardiac, hematologic and psychiatric diseases. All patients signed an informed consent prior to the study. Two large centers in Saudi Arabia were involved and ethical approval was obtained from the local research ethics committees in both centers.

Patients were treated with either PEG-IFN α-2b (1.5 μg/kg/wk) or PEG-IFN α-2a (180 μg/wk) and ribavirin (13.3 mg/kg/day).

The demographic variables of age, sex and educational status were collected. Before recruitment, all patients were also interviewed and examined by a qualified ear nose and throat (ENT) physician.

The pre-treatment pure tone audiogram (PTA), tympanogram and distortion-product otoacoustic emissions (DPOAE) were performed and these procedures were repeated three months after starting therapy and three months after discontinuation of therapy.

### Audiology

Routine air-conduction PTA (0.125, 0.25, 0.5, 1, 2, 3, 4, 6, and 8 kHz) was carried out under standardized audiometric conditions in a sound-attenuating proof test booth. Clinically significant hearing changes were determined based on criteria from the American Speech-Language-Hearing Association[23] which include 20 dB threshold shift at a single frequency, 10 dB shift at two adjacent frequencies or loss of response at three adjacent frequencies. Since INF can cause flu-like symptoms that may interfere with Eustachian tube function, typanogram was performed at the same time with PTA.

Pre-treatment DPOAE testing was also conducted. This test provides a noninvasive, objective measure of cochlear function. DPOAE is an acoustic response generated by the outer hair cells within the cochlea and reverse transmitted through the middle-ear into the ear canal. OAEs have been used widely for ototoxicity monitoring in pediatric populations receiving ototoxic medications in which OAE changes tend to occur before conventional frequency pure-tone threshold changes[24,25]

All statistical analyses were performed with the Statistical Package for the Social Sciences 11.0 (SPSS, Chicago, IL). Student t-test was used for quantitative parametric variables evaluations between two groups. A *P* value of <0.05 was considered to be statistically significant.

## RESULTS

The enrollment criteria were met by 21 patients with chronic HCV. All patients finished a full course of pegylated interferon plus ribavirin therapy for genotype 4 hepatitis C which is 36-48 weeks with no side effect related treatment withdrawals. The interferon dose was maintained at the same level throughout the treatment duration. Ribavirin was reduced transiently in two patients because of anemia and both patients received erythropoietin a at a dose of four thousand units twice a week for a month.

The mean age was 45.7 years. The male to female ratio was 1.1:1. The mean PTA was 15.9 ± 5.3 before treatment, 17.4 ± 6.1 during treatment, and 16.5 ± 5.1 after treatment. The differences between pre and mid, pre and post, and mid and post-treatment PTA were not significantly different (*P*>0.05) [Table 1]. We also compared the mean PTA for individual ears: PTA was 15.9 ± 5.7 before treatment, 17.4 ± 6.6 during treatment and 16.5 ± 6.3 after treatment. There were no significant differences in the PTA during the various study periods (*P*>0.05) [Table 2].

**Table 1:** Comparing pre, mid and post PTA (*n*=21)

	Pre	Mid	Post
Mean	15.9	17.4	16.5
SD	5.3	6.1	5.1

PTA: Pure-tone audiometry. Pre *vs*. mid (*t*=1.55 and *P*=0.137), pre *vs*. post (*t*=0.50 and *P*=0.623) and mid *vs*. post (*t*=0.69 and *P*=0.35).

**Table 2:** Comparing pre, mid and post PTA (ear) (*n*=42)

	Pre	Mid	Post
Mean	15.9	17.4	16.5
SD	5.7	6.6	6.3

PTA: Pure-tone audiometry. Pre *vs*. mid (*t*=1.99 and *P*=0.054), pre *vs*. post (*t*=0.61 and *P*=0.543) and mid vs. post (*t*=1.09 and *P*=0.284).

In comparing the tympanograms for each individual ear, the tympanogram type C pattern constituted 4.8% of the population before treatment, 4.8% during treatment and 7.1% after treatment. The differences between pre and mid, pre and post and mid and post were not statistically significant (*P*>0.05) [Table 3].

**Table 3:** Comparing pre, mid and post tympanogram (ears) (*n*=42)

	Pre		Mid		Post	
	No.	%	No.	%	No.	%
A	40	95.2	40	95.2	39	92.9
C	2	4.8	2	4.8	3	7.1
Total	42	100	42	100	42	100

Pre *vs*. mid *P*>0.05, pre *vs*. post *P*>0.05 and mid *vs*. post (*P*>0.05)

Finally, we compared the presence of DPOAE (P) for all patients. DPOAE (P) constituted 85.7% before treatment, 90.5% during treatment and 88.1% after treatment. The differences between pre and mid, pre and post and mid and post-treatment DPOAE were not significantly different (*P*>0.05) [Table 4].

**Table 4:** Comparing pre, mid and post OAE (ears) (*n*=42)

	Pre		Mid		Post	
	No.	%	No.	%	No.	%
A	6	14.3	4	9.5	5	11.9
P	36	85.7	38	90.5	37	88.1
Total	42	100	42	100	42	100

OAE: Otoacoustic emission, Pre *vs*. mid *P*>0.05, pre *vs*. post *P*>0.05, and mid *vs*. post *P*>0.05,

## DISCUSSION

There are over 130 medicinal and chemical agents with potential for damaging the cochlear and/or vestibular end-organs.[26] Life-threatening medical conditions may require treatment with highly ototoxic agents and the risk of hearing loss may be unavoidable. In many situations, however, alternative drugs, reduced dosages, or altered treatment regimens are options if ototoxicity is detected early in the treatment period.

In general, predicting the occurrence of ototoxic hearing loss in any clinical situation is a clinical challenge. The risk for developing hearing loss from ototoxic medication is generally correlated with dosage, although this relationship is highly variable.[27] However, individual susceptibility to ototoxic hearing loss is influenced by multiple biochemical, physiologic, and genetic factors.[28] These effects usually begin near the high-frequency encoding cochlear basal region and progresses toward the apex of the cochlea.[29–31] Thus, the hearing changes do not occur in the frequencies required for proper speech reception. Therefore, even with medications well known for their ototoxic potential, the patients’ self reporting of symptoms presents a clinical challenge in diagnosing hearing loss, even more so with unilateral hearing loss. Hence, a direct measurement of hearing is essential to truly capture hearing-related effects.

Little is known about the individual drug sensitivity of the inner ear in patients receiving PEG-IFN\RBV treatment. Recently, several studies have been published suggesting a possible role of interferon in attenuating hearing loss; however, these studies have been largely limited to case reports and animal studies.

In our prospective study, we have demonstrated that HCV patients receiving PEG-IFN\RBV treatment did not experience any significant hearing loss (sensorineural, conductive or both) using sensitive and objective measures before, during, and after therapy. Two recently published studies performed in patients with hepatitis B have come to differing conclusions, Kaygusuz *et al*.[32] did not find any negative effects of IFN in hepatitis B patients, while Gorur *et al*. demonstrated significant hearing loss in their cohort.[33]

Although, reported cases of sensorineural hearing loss caused by IFN occurred when used in combination with RBV, the role of RBV in the development of sensorineural hearing loss in those patients is unclear. To date, there are no published reports of hearing loss due to RBV monotherapy.

Many reported cases of PEG-IFN ototoxicity experienced sudden sensorineural hearing loss (SSNHL), rather than a gradual decline in hearing. The reasons for selective involvement of the cochlear and rarely vestibular[34] functions is unknown. SSNHL *per se* remains controversial in many aspects. The definition itself is difficult to apply when a study is retrospective in nature, because for most patients, the hearing level before the onset of SSNHL is unknown, so assigning it as 0 dB (or the same as the unaffected ear) carries some error. Moreover, many patients who recovered spontaneously soon after the onset of SSNHL do not seek medical help. In addition, the cause or mechanism of this entity is unknown. One of the theories to explain this type of hearing loss is an autoimmune mechanism[8] which can be induced by IFN. In general, autoimmune sensorineural hearing loss (ASNHL) is believed to be the most common cause of sudden hearing loss in adults,[35] but this is unlikely in the case of IFN as most cases reported only unilateral hearing loss. On the other hand, the possibility of a preexisting hearing loss as a form of an extrahepatic manifestation of hepatitis C cannot be excluded. Formal auditory testing was not performed prior to therapy in these studies, and as such the condition may have existed previously but gone unnoticed by the patient initially.[36]

A second hypothesis for hearing loss is peripheral neurotoxicity of PEG-IFN.[37,38] However, high-frequency sensorineural hearing loss and absent responses in distortion product otoacoustic emissions are clear indicators of cochlear site of toxicity.[18] A third possibility raised by some authors is transient ischemia in one ear induced by IFN. This may explain why most patients recover after cessation of treatment.[8,10,15]

Akyol *et al*. performed a randomized study to prospectively investigate the possible ototoxic effects of IFN-a 2A in an animal (mouse) model.[19] In these mice, there was no loss of hair cells (a major histopathologic feature linked to ototoxic agents, such as aminoglycosides), but rather they found a histopathologic picture similar to the one associated with salicylate ototoxicity, which is known to be mild and reversible.

The main strengths of the current study are that it used detailed and objective measures of hearing in a prospective fashion. These tests were done before, during, and after therapy clearly enabling us to detect pre-existing hearing abnormalities, timing of IFN ototoxicity, if any, and reversibility of any possibly detected abnormalities. However, no such toxicity was observed. Nonetheless, our study is limited by a small sample size since a larger sample size would probably be required for an uncommon effect to be demonstrated.
